# Special Issue on the 60^th^ anniversary of the first laser—Series I: Microcavity Photonics—from fundamentals to applications

**DOI:** 10.1038/s41377-021-00583-w

**Published:** 2021-07-08

**Authors:** Yun-Feng Xiao, Frank Vollmer

**Affiliations:** 1 State Key Laboratory for Mesoscopic Physics and Frontiers Science Center for Nano-optoelectronics, School of Physics, Peking University, Beijing, China; 2 Collaborative Innovation Center of Extreme Optics, Shanxi University, Taiyuan, China; 3 Department of Physics and Astronomy, Living Systems Institute, University of Exeter, Exeter, UK

**Keywords:** Lasers, LEDs and light sources, Other photonics

Optical microcavities confine light to small volumes by resonant recirculation. Because of their ultrahigh quality factors (*Q*) and small mode volumes (*V*_*m*_), optical microcavities have attracted strong research interests for their unique property of significantly enhancing light–matter interaction.^1^ In recent decades, optical microcavities have become cornerstones for a wide range of studies and applications, including nonlinear photonics^2–4^, non-Hermitian^5,6^ and chaotic physics^7,8^, cavity quantum electrodynamics^9,10^ and cavity optomechanics^11^, optical sensing^12,13^, and in particular, *microlasers*^14,15^. Optical cavities serve as an essential building block of lasers from the date of their birth by enhancing photon–material interactions and providing optical feedbacks. In the past decades, along with the development of various nanofabrication and packaging techniques, microcavities with ultrahigh quality factors and miniatured sizes have enabled low-threshold and high-coherence lasers down to chip scale. Meanwhile, laser physics and applications have been greatly advanced by exploiting the spectral, temporal, and spatial degrees of freedom of microcavities and their rich interactions with various materials. Nowadays, microcavity-based lasers provide not only indispensable tools in applied researches such as biochemical sensing/imaging and high precision spectroscopy, but also practical devices including atmospheric monitors or smart phone and computer chips that have the potential for entering diverse aspects of people’s daily life.

This special issue covers a series of cutting-edge works on advanced physics and applications of optical microcavities and microlasers, ranging from the study of chaotic resonances, microcombs and soliton physics, lasers with tailored orbital angular momentum, coherent light-matter coupling and quantum condensation, optical nonreciprocity, to multiplexed biochemical sensing. In what follows, a brief introduction to each topic will be presented along with their key implications highlighted.

Observation and understanding of the cavity mode structure and dynamics is the foundation of its engineering and applications. While numerous experimental techniques have been established to understand the microcavity internal wave dynamics based on the input-output characteristics, direct inspection into the black box is always highly desired yet challenging, especially regarding the complex chaotic electromagnetic fields of asymmetric microcavities. Writing in this special issue, Wang, S. et al. demonstrate a simple but robust approach to directly and rapidly map the internal mode patterns in chaotic microcavities^16^. With this technique, chaos-assisted tunneling and its time-reversed process are experimentally observed in the optical domain with unprecedented certainty. This research offers a new pathway in the understanding of the ultrafast and delicate physical processes in optical microcavities.

The interplays between optical nonlinearity and dispersion, loss and gain give rise to an important branch in microcavity photonics, i.e., the optical frequency combs, and especially, the dissipative Kerr solitons (DKSs). DKSs are on-chip coherent light sources with equidistant frequency lines spanning a wide spectral range, which find widespread applications in microwave source generation, optical spectroscopy, and precision measurements. This special issue brings up a collection of original research works in the field of soliton fundamentals and applications. Wang, H. et al. demonstrated the peculiar characteristics of Dirac solitons in microresonators, a new type of soliton in microresonators resulting from broadband modal coupling, by solving the corresponding conservative coupled Lugiato-Lefever equation (LLE) non-perturbatively and using the exact solution as a soliton ansatz for the hybrid system^17^. The properties of Dirac optical solitons in microresonators are important at a fundamental level and provide a road map for soliton microcomb generation in the visible band. Application-wise, Wang, B. et al. established a new high-power, high coherence photonic millimeter-Wave (mmWave) platform through the combination of integrated microresonator solitons and high-speed photodiodes^18^. Importantly, the power level with microresonator solitons approaches the theoretical limit of heterodyne detection, which assumes an ideal photodiode with zero power roll-off in its frequency response, providing a viable path to chip-scale, high-power, low-noise, and high-frequency sources for mmWave applications. Qin et al. reported the realization of electrically controllable laser frequency combs in graphene-fiber microresonators with unprecedented dynamic tunability^19^. Such realization of the dynamic control and stabilization of the microcomb in a heterogeneous graphene-fiber microcavity provides a new platform for the interfacing of single-atomic-layer optoelectronics and ultrafast photonics, and will promote versatile applications for arbitrary waveform generation, fiber communication, signal processing, and spectroscopic metrology.

Orbital angular momentum (OAM) is another important degree of freedom of light that has drawn a great deal of interests recently. Besides their novel interaction physics with matters, OAM-carrying lights can in principle support infinite orthogonal optical modes that are feasible for high-capacity optical communication and information processing. Whispering gallery microcavities are a natural platform for generating and studying OAM light, with their azimuthal mode number directly correlated to the OAM number. In this special issue, Zhang et al.^20^ experimentally demonstrated fast control of the fractional orbital angular momentum (FOAM) from a vortex semiconductor microlaser based on fast transient mixing of integer laser vorticities induced by a control pulse, which are highly desirable for OAM applications. On the other hand, Wang, J. et al.^21^ explored OAM physics in exciton-polaritons, a coherent light-matter coupled system. As Bosonic quasiparticles, exciton-polaritons undergo spontaneous quantum condensation above the threshold, generating coherent light which is also termed as “polariton lasers”. By engineering artificial annular potential landscapes in halide perovskite semiconductor microring cavities, they experimentally and theoretically investigated room-temperature condensation of exciton-polariton orbital states with symmetric petal-shaped patterns in real space, resulting from symmetry breaking due to the anisotropic effective potential of the birefringent perovskite crystals. This work thus demonstrates the feasibility of precisely control the OAM of light by manipulating both the light and matter degrees of freedom in a strongly coupled system.

Optical nonreciprocity is critical for optical communication and information processing. Optical nonreciprocal systems enforce one-way transmission of the light signals, which requires the breaking of the Lorentz reciprocity theorem. To date, the realization of optical nonreciprocity generally requires time-reversal symmetry breaking via a magnetic field or magnetic order, or nonlinear optical effects, which requires either complex fabrications, stringent experimental conditions, or high power consumptions. In this special issue, Huang et al.^22^ took a step towards solving these issues, by proposing an magnetic-field-free nonreciprocal system in the linear optical regime. The authors took advantages of energy loss, which is usually regarded as harmful, to generate optical nonreciprocity. The energy loss introduces a phase lag in a resonance mode, which, in an optical resonator network, gives rise to different interference conditions in the forward and backward propagation directions, and hence leading to nonreciprocal transmission. This work provides a general approach that may open new possibilities in designing nonreciprocal devices.

Optical microcavities manifest themselves by virtue of the strongly enhanced light–matter interaction, and thus have witnessed tremendous progresses in ultrasensitive detection of nanoparticles, biomolecules, and physical quantities (temperature, force, pressure, electric and magnetic fields) over the past decades. However, most existing microsensors were enabled by tracking a single optical mode, which limits its multiplexing capabilities. Nowadays, the field is developing towards more sophisticated sensing functionalities such as specific identifications of mixed particles, real-time monitoring of complex physical processes, and in-situ biochemical imaging, where it is necessary to take advantages of the spectral degree of freedom. This special issue includes three innovative works demonstrating state-of-the-art sensing techniques toward this direction. Tang et al.^23^ developed wavelength-encoded microdisk lasers with omnidirectional emission as imaging probes to track the real-time movements of live cells. The omnidirectional laser emission was achieved by introducing boundary defects or scattering layers into microcavity designs. Transferred into live cells in vitro, the omnidirectional laser particles allow massive multiplexed tagging of single cells and achieved continuous tracking of moving cells with high signal-to-noise ratios for 2 h, which opens the avenue to analyze live cell activities and heterogeneous cell populations. In ref. ^24^, Liao et al. demonstrated an optical WGM barcode technique involving simultaneous monitoring of the patterns of multiple modes for temperature sensing. By cross-referencing the different responses of different modes to the environmental temperature, one-shot measurement is made possible without the information of the initial state, which ensures high-precision and wide-range measurement of actual temperature. In another work, Yang et al.^25^ demonstrated operando monitoring of the transition dynamics of a phase-change material via a self-referencing optofluidic microcavity. A pair of cavity modes are used to precisely decouple the refractive index and temperature information of the analyte during the phase-transition process.

To highlight the inspiring advances of the applications of microcavity lasers in biosensing, in this special issue, NikToropov et al.^26^ provided an comprehensive review on recent progresses in biosensing with whispering-gallery mode lasers. This review covers the basic concepts of WGM resonators, the integration of gain media into various active WGM sensors and devices, and the cutting-edge advances in photonic devices for micro- and nanoprobing of biological samples that can be integrated with WGM lasers.

In summary, this special issue focuses on microcavities and microlasers, aiming at advancing the fundamental understanding and applications in microcavity photonics by exploring diverse degrees of freedom. We appreciate very much the great efforts and contributions of all authors, and hope that this special issue will provide a good reference as well as fuel the inspiration for researchers in this promising field of microcavity photonics, and significant progress is still anticipated to be achieved with more scientific groups entering this field.

## References

[1] Xiao, Y.-F., Zou, C.-L., Gong, Q., Yang, L. *Ultra-High-Q Optical Microcavities*. World Scientific: 2020.

[2] Lin G, Coillet A, Chembo YK (2017). Nonlinear photonics with high-Q whispering-gallery-mode resonators. Adv. Opt. Photon..

[3] Strekalov DV, Marquardt C, Matsko AB, Schwefel HG, Leuchs G (2016). Nonlinear and quantum optics with whispering gallery resonators. J. Opt..

[4] Picqué N, Hänsch TW (2019). Frequency comb spectroscopy. Nat. Photon.

[5] Wiersig J (2014). Enhancing the sensitivity of frequency and energy splitting detection by using exceptional points: application to microcavity sensors for single-particle detection. Phys. Rev. Lett..

[6] Chen W, Kaya Özdemir Ş, Zhao G, Wiersig J, Yang L (2017). Exceptional points enhance sensing in an optical microcavity. Nature.

[7] Cao H, Wiersig J (2015). Dielectric microcavities: model systems for wave chaos and non-Hermitian physics. Rev. Mod. Phys..

[8] Jiang X (2017). Chaos-assisted broadband momentum transformation in optical microresonators. Science.

[9] Kimble HJ (2008). The quantum internet. Nature.

[10] Lu Y-K (2018). Spontaneous T-symmetry breaking and exceptional points in cavity quantum electrodynamics systems. Sci. Bull..

[11] Aspelmeyer M, Kippenberg TJ, Marquardt F (2014). Cavity optomechanics. Rev. Mod. Phys..

[12] Foreman MR, Swaim JD, Vollmer F (2015). Whispering gallery mode sensors. Adv. Opt. Photon..

[13] Zhi Y, Yu X-C, Gong Q, Yang L, Xiao Y-F (2017). Single nanoparticle detection using optical microcavities. Adv. Mater..

[14] He L, Özdemir ŞK, Yang L (2013). Whispering gallery microcavity lasers. Laser Photonics Rev..

[15] Jiang X-F, Zou C-L, Wang L, Gong Q, Xiao Y-F (2016). Whispering-gallery microcavities with unidirectional laser emission. Laser Photonics Rev..

[16] Wang S (2021). Direct observation of chaotic resonances in optical microcavities. Light Sci. Appl..

[17] Wang H (2020). Dirac solitons in optical microresonators. Light. Sci. Appl..

[18] Wang B (2021). Towards high-power, high-coherence, integrated photonic mmWave platform with microcavity solitons. Light.: Sci. Appl..

[19] Qin C (2020). Electrically controllable laser frequency combs in graphene-fibre microresonators. Light.: Sci. Appl..

[20] Zhang Z (2020). Ultrafast control of fractional orbital angular momentum of microlaser emissions. Light.: Sci. Appl..

[21] Wang J (2021). Spontaneously coherent orbital coupling of counterrotating exciton polaritons in annular perovskite microcavities. Light.: Sci. Appl..

[22] Huang X (2021). Loss-induced nonreciprocity. Light: Sci. Appl..

[23] Tang S-J (2021). Laser particles with omnidirectional emission for cell tracking. Light.: Sci. Appl..

[24] Liao J, Yang L (2021). Optical whispering-gallery mode barcodes for high-precision and wide-range temperature measurements. Light.: Sci. Appl..

[25] Yang D-Q (2021). Operando monitoring transition dynamics of responsive polymer using optofluidic microcavities. Light.: Sci. Appl..

[26] Toropov N (2021). Review of biosensing with whispering-gallery mode lasers. Light.: Sci. Appl..

